# Factors associated with depression in patients with Parkinson’s disease A multicenter study in Lima, Peru

**DOI:** 10.1590/1980-57642018dn12-030010

**Published:** 2018

**Authors:** Nilton Custodio, Carlos Alva-Diaz, Cristian Morán-Mariños, Koni Mejía-Rojas, David Lira, Rosa Montesinos, Eder Herrera-Pérez, Sheila Castro-Suárez, Yadira Bardales

**Affiliations:** 1 Department of Neurology, Peruvian Institute of Neurosciences, Lima, Peru.; 2 Diagnostic Unit for Cognitive Impairment and Dementia Prevention, International Clinic, Lima, Peru.; 3 Research Unit, Peruvian Institute of Neurosciences, Lima, Peru.; 4 Clinical and Healthcare Efficiency Network (REDECS). Lima, Peru.; 5 Neurology Department, Hospital Nacional Daniel Alcides Carrión, Callao, Peru.; 6 Research Center OADI, Hospital Nacional Daniel Alcides Carrión, Callao, Peru.; 7 Peruvian Student Medical Scientific Society, Universidad Privada San Juan Bautista (SOCIEM - UPSJB). Lima, Peru.; 8 Rehabilitation Medicine Department, Peruvian Institute of Neurosciences, Lima, Peru.; 9 Research and Teaching Unit. International Clinic. Lima, Peru.; 10 Department of Neurology of Human Behavior. National Institute of Neurological Sciences. Lima, Peru.; 11 Department of Neurology, International Clinic. Lima, Peru.

**Keywords:** Parkinson’s disease, depression, risk factors, doença de Parkinson, depressão, fatores de risco

## Abstract

The diagnosis and treatment of depression in patients with Parkinson’s disease (PD) is inadequate, often contributing to a reduced quality of life, rapid disease progression, higher cognitive impairment, and an increased burden of care for family members of patients with PD.

**Objective:**

To determine the factors associated with depression in PD and to examine the frequency of depressive symptoms among patients with PD.

**Methods:**

This study was an observational, analytical, multicenter study of a cross-sectional cohort, conducted between July 2016 and May 2017. PD patients were recruited from neurology clinics in Lima, Peru. All statistical analyses were performed using descriptive statistics. Bivariate and multivariate logistic regression analyses were calculated using STATA.

**Results:**

Out of 124 patients (average age: 68.7 years; 58% males) included in the study 60.5% (75/124) presented with symptoms of depression; only 20% (25/124) received antidepressants. Factors associated with depression in PD included: unemployment, falls, freezing of gait, involuntary movements micrographia, stooped posture, hyposmia, movement disorders in sleep, rapid disease progression, and the use of MAOIs. Furthermore, statistically significant differences were found in disease duration, UPDRS and MMSE scores, Hoehn and Yahr (HY) stage, and length of time taking L-dopa between PD patients with and without depressive symptoms.

**Conclusion:**

Factors associated with depressive symptoms in patients with PD were hyposmia, rapid progression of the disease, the use of L-dopa, and use of MAOIs. The frequency of depressive symptoms in patients with PD is high; early diagnosis and prompt treatment are needed to improve their quality of life and the family environment.

## Resumo

O diagnóstico e tratamento da depressão em pacientes com doença de Parkinson (DP) é inadequado, frequentemente contribuindo para a redução da qualidade de vida, progressão rápida da doença, maior comprometimento cognitivo e aumento da carga de cuidado aos familiares de pacientes com DP.

**Objetivo:**

Determinar os fatores associados à depressão na DP e examinar a frequência de sintomas depressivos entre pacientes com DP.

**Métodos:**

Este estudo foi um estudo observacional, analítico, multicêntrico, de uma coorte transversal conduzida entre julho de 2016 e maio de 2017. Os pacientes com DP foram recrutados em clínicas de neurologia em Lima, Peru. Todas as análises estatísticas foram realizadas por meio de estatística descritiva. Análises de regressão logística bivariada e multivariada foram calculadas usando STATA.

**Resultados:**

Dos 124 pacientes (idade média: 68,7 anos; 58% homens) incluídos no estudo, 60,5% (75/124) apresentaram sintomas de depressão; apenas 20% (25/124) receberam antidepressivos. Os fatores associados à depressão na DP incluíram: desemprego, quedas, congelamento da marcha, movimentos involuntários micrografia, postura inclinada, hiposmia, distúrbios do movimento no sono, progressão rápida da doença e uso de inibidores da MAO. Além disso, houve diferenças estatisticamente significativas encontradas em: duração da doença, escores nas escalas UPDRS e MMSE, estágio Hoehn e Yahr (HY) e tempo de duração da L-dopa entre os pacientes com DP, entre aqueles com e sem sintomas depressivos.

**Conclusão:**

Fatores associados a sintomas depressivos em pacientes com DP foram hiposmia, rápida progressão da doença, uso de L-dopa e uso de IMAOs. A frequência de sintomas depressivos em pacientes com DP é alta; o diagnóstico precoce e o tratamento imediato são necessários para melhorar a qualidade de vida e o ambiente familiar.

Parkinson’s disease (PD) is the second-most-common neurodegenerative disorder after Alzheimer’s disease;^1^^,^ 2 yet there is insufficient research on non-motor symptoms of PD, such as depression and anxiety. Depression, a comorbidity in PD, is commonly associated with cognitive impairment and may precede motor symptoms; it is a prevalent condition in newly diagnosed patients with PD.^3^ The prevalence of PD in industrialized countries ranges from 0.3% of the general population and 1% in persons over 60.^4^ In the European study conducted by De Rijk et al.,^5^ the rates ranged from 100-200 per 100,000 inhabitants with an annual incidence of 13 cases per 100,000 inhabitants reported. The prevalence of PD in Peru is unknown, but according to the Bureau of Statistics and Informatics of the National Institute of Neurological Sciences (INCN),^6^ there were 670 PD cases notified in 2013, representing the ninth leading cause of morbidity in outpatient visits and 2.6% of medical visits conducted.

Recent studies indicate that depression is a manifestation that precedes the motor symptoms exhibited in PD. Epidemiological studies have shown that depression in PD is greatest in patients who suffer from other neurological disorders,^6^ it increases executive dysfunction,^7^ is associated with chronic pain^8^ and loss of pleasure.^9^ Depression in PD is also associated with more severe cognitive and functional alterations, as well as more rapid disease progression, decreased quality of life,^10^ increased morbidity and mortality, increased hospitalization,^11^ and creates a burden for caregivers.^12^ It is estimated that around half of these patients do not receive treatment for depression.^13^

There are currently 9 screening scales to assess depression, of these, the Hamilton Depression Rating Scale (HAM-D) is one of the most widely used.^13^ However, there is a greater tendency for research studies to use Beck’s Depression Inventory (BDI) within the PD patient population as it has demonstrated higher rates of reliability in comparison to other scales.^14^^-^ 16

Depression and PD share several pathophysiological aspects. In patients with PD, the progressive loss of striatal dopaminergic innervation damages the cortical limbic regions,^17^ while depression may be due to damage of the locus coeruleus complex and the interruption of the serotonergic afferent pathways as observed from stage 2 of PD. Another overlapping symptom between both illnesses is the decreased serotonergic activity found in the cerebrospinal fluid and brains of patients with PD. Therefore, the most accepted hypothesis for the development of depression in PD are alterations in the dopaminergic and serotonergic pathways,^17^ as evidenced by neuroimaging studies showing loss of brain tissue in the cortical-limbic network.

The frequency of depression in PD varies across studies according to the population evaluated and diagnostic instruments used. The reported frequencies vary widely from 2.7% to more than 90%.^18^

Some studies have found various factors associated with depression in PD, including cognitive impairment,^3^^,^ 19 bradykinesia,^20^ primarily affecting the dominant side,^21^ longer duration of PD,^19^ and rapid progression of symptoms. With regard to other factors associated with depression in PD, such as age, gender, disease duration, clinical presentations, and antiparkinsonian agents, reports are conflicting.^19^

The main objective of this study was to determine the factors associated with symptoms of depression in patients with PD in neurological centers of Peru. Secondary objectives were to determine the frequency of depression in patients with PD and describe epidemiological, clinical and pharmacological features of PD patients exhibiting symptoms of depression.

## METHODS

The study utilized an analytical multicenter cross-sectional design. The study population comprised all PD patients treated at the Peruvian Institute of Neurosciences in Lima and at different neurology hospitals in Peru. Sample size estimation was done with level of significance set at 5%, study power at 80%, and parameters derived from the study by Pinto et al.^22^ The estimated sample size was approximately 45 patients per group (with and without depressive symptoms). The unit of analysis was all patients with PD seen at the Peruvian Institute of Neurosciences in Lima, the International Clinic, and the Department of Neurology at Hospital Nacional Daniel Alcides Carrión. Non-probability convenience sampling was employed. Qualitative variables were analyzed using frequencies and percentages, while measures of central tendencies and dispersion were computed for quantitative variables. Bivariate analysis comprised Chi-square tests for qualitative variables and Student’s *t*-test or non-parametric tests for quantitative variables, depending on the Kolmogorov-Smirnov tests for normality of data. STATA statistical software version 14 (StataCorp LP, College Station, TX, USA) was used for statistical analysis. Inclusion criteria consisted of: a diagnosis of PD without cognitive impairment as per the United Kingdom Parkinson Disease Society Brain Bank listed criteria; having been treated at the International Clinic of the Department of Neurology & Neurorehabilitation of the Peruvian Institute of Neuroscience, or the Department of Neurology at the Hospital Nacional Daniel Alcides Carrión between July 2016 and May 2017; patients over 18 years of age; an informed consent signed by either the patient or family members on his/her behalf. Exclusion criteria consisted of: a diagnosis of PD with a level of cognitive impairment that precluded the application of Beck’s Depression Inventory (BDI-II); a Mini-Mental State Examination (MMSE) score of less than 25.^23^ Data collection was carried out using a structured interview where data on sociodemographic variables were collected including age, gender, level of education completed, place of origin, and race; clinical factors such as age at onset of PD, time of disease progression, clinical features of PD, symptoms of PD, the presence of non-motor symptoms in PD, rapid progression of PD motor symptoms, the UPDRS and MMSE scores, Hoehn and Yahr (HY) stage; and pharmacological factors, such as the type of medication used (levodopa, dopaminergic agonists - pramipexole, biperiden and MAOIs-rasagiline). In order to assess motor and non-motor symptoms (falls, asymmetrical limb rigidity, freezing of gait, movement disorders in sleep, micrographia, walking difficulties, stooped posture, visual hallucinations, hypophonia, urinary urgency, constipation, and rapid disease progression) a questionnaire was applied in such a way that symptoms were noted either as previous patient history or as first prodromal symptom; however, this was not measured objectively. Symptoms of depression were determined by the BDI-II. Both MMSE and BDI-II have been validated in Spanish versions and previously employed in our study setting.^24^

The present study was executed with strict adherence to ethical codes; patients were not subject to any intervention. This study was approved by the local Research Ethics Committee of the Universidad San Martin de Porres. Informed consent was obtained from participant or next-of-kin.

## RESULTS

A total of 123 patients met the inclusion criteria, of which 75 (60.9%) had symptoms of depression according to BDI-II. Among these patients, the degree of more frequent depression was moderate (26.7%). Only 20.6% of patients were taking antidepressants. 45% of patients with PD exhibiting depression had insomnia and 19.1% had suicidal ideations (Table 1).

**Table 1:** Depressive symptom profile in patients with PD.

Variables		n (%)
BDI results	With depressive symptoms	75 (60.9)
	No depressive symptoms	48 (39.0)
BDI degree of depression	Mild	28 (22.7)
	Moderate	33 (26.7)
	Severe	13 (10.5)
Use of antidepressant medications	Yes	25 (20.66)
	No	96 (79.34)
Insomnia	Yes	54 (45.00)
	No	66 (55.00)
Suicidal ideation	Yes	23 (19.01)
	No	98 (80.99)
Psychiatric history	Yes	7 (5.79)
	No	114 (94.21)

BDI-II: Beck Depression Inventory.

Patients with PD and depressive symptoms had an average age of 69 years, 58.6% were male, most were married, highly educated, unemployed, born in the coastal region of Peru and were residents of Lima. Only unemployment was correlated with symptoms of depression (Table 2).

**Table 2:** Epidemiological characteristics in patients with PD.

		Depressive symptoms		p
		Yes (%)	No (%)	
Age (95% CI)		69.06 (66.90-71.23)	68.29 (64.87-71.70)	0.687+
Gender	Male	44 (58.6)	27 (56.2)	0.791*
	Female	31 (41.3)	21 (43.7)	
Marital status	Single	4 (5.33)	2 (4.1)	0.179**
	Married	61 (81.3)	35 (72.9)	
	Divorced	0	3 (6.2)	
	Widowed	10 (13.3)	8 (16.6)	
Education level	Low Level (elementary school)	11 (14.6)	5 (10.4)	0.494*
	High Level (high school)	64 (85.3)	43 (89.5)	
Current occupation or status	Employed	18 (24)	25 (52.0)	0.001*
	Unemployed	23 (47.9)	57 (56)	
Region of birth	Coastal region	42 (56.7)	26 (56.5)	0.358**
	Highland region	31 (41.8)	17 (36.9)	
	Jungle region	1 (1.3)	3 (6.5)	
Place of residence	Lima	63 (84)	39 (81.2)	0.436**
	Province	12 (16)	9 (18.7)	

* Value calculated using Chi-square test.

** Value calculated using Fisher’s exact test.

+ Value calculated using Student’s *t*-test.

Clinical characteristics of patients with PD associated with depression included: disease duration, falls, freezing of gait, involuntary movements (micrographia, kyphosis), hyposmia, movement disorders in sleep, and rapid disease progression (p<0.05). Other characteristics significantly associated with patients suffering from depression included having a HY stage ≥2.5, a UPDRS score >41, pharmacological characteristics of long-term use of L-dopa (in years) and the use of MAOIs-rasagiline (Table 3). Notwithstanding, when the multivariate analysis (logistic regression) was performed, the clinical factors that continued to be associated were hyposmia and rapid progression of disease; as well as both pharmacological factors (long-term use of L-dopa >4 years, and use of MAOIs-rasagiline) (Table 3).

**Table 3:** Clinical and pharmacological characteristics of PD patients with depression.

	n (%)	p		n (%)	p
Onset of symptoms (Me;IQR)	63 (55-68)	0.36+	Visual hallucinations	11 (15.07)	0.148*
Disease duration (Me;IQR)	6;3-9	0.0001±	Hypophonia	57 (78.08)	0.648*
Clinical presentations			Urinary urgency	58 (79.45)	0.099*
• Rigidity	40 (53.33)	0.558*	Hyposmia	53 (72.60)	0.558* ++
• Trembling	35 (46.67)		Constipation	42 (57.53)	0.169*
Initial symptoms			Rapid progression	9 (12.33)	0.012* ++
• Tremors	38 (50.67)	0.942*	H&Y (Me;IQR)	2.5;2-3	
• Akinetic-rigid syndrome	37 (49.33)		• <2.5	20 (26.67)	0.942*
Predominant symptom			• ≥2.5	55 (73.33)	
• Tremors	30 (41.67)	0.610*	UPDRS III (Me;IQR)	59; 47-68	
• Akinetic-rigid	42 (58.33)		• ≤40	18 (37.5)	0.02*
Hemibody involved			• >41	30 (62.5)	
• Right	50 (66.67)	0.636*	MOCA (Me;IQR)	27;7-30	0.467+
• Left	25 (33.33)		Pharmacological treatment		
Motor and non motor			• Time using L-DOPA		< 0.000* ++
• Falls	28 (38.36)	0.026*	< 3 years	37 (46.3)	
• Asymmetric stiffness	69 (94.52)	0.565**	≥4 years	34 (24.7)	
• Freezing of gait	32 ( 43.84)	0.001*	• L-DOPA	67 (63.3)	0.059*
• Myoclonus	49 (67.12)	0.027*	• Dopamine agonists	33 (45.21)	0.327*
• Micrographia	59 (80.82)	0.006*	• Anticholinergics	11 (8.7)	0.183*
• Abnormal gait	54 (73.97)	0.098*	• MAOI	33 (45.21)	0.006* ++
• Kyphosis	58 (79.45)	0.034*			

Me: Median. IQR: Interquartile Range. H&Y: Hoehn & Yahr stage. MAOI: Monoamine Oxidase Inhibitor. UPDRS III: Unified Parkinson’s Disease Rating Scale – motor section. MOCA: Montreal Cognitive Assessment.

* Value calculated using Chi-square test.

** Value calculated using Fisher’s exact test.

+ Value calculated using Student’s *t*-test.

++ Study will be conducted using logistic regression.

The predictive ability (R2) of the regression model improved from 23.5% to 27.2% when MAOIs-rasagiline was included.

## DISCUSSION

This study found that the frequency of symptoms of depression in patients with PD was 60.9%, similar to that reported in other investigations: 54% in the study carried out by Pinto et al.,^22^ 50% in the study of Serrano-Dueñas,^21^ and 48.8-50.1% as reported by Chaudhuri et al.^25^ These three studies found that non-motor symptoms of PD were the most under-estimated and least researched, despite being major determinants of health-related quality of life.^26^

The frequency of depressive symptoms in PD in the present study was lower than the rates reported by Saints in a Spanish population (68%),^27^ or Cosentino et al. who reported a prevalence of 80.7% in a Peruvian population at INCN, albeit having used a different instrument (PDNMSQuest).^28^ However, our results were higher than those reported by Chaná-Cuevas et al.,^29^ who reported a frequency of 24.7% having also used the BDI, or Wichowicz et al., who found a rate of 35%, among other authors.^30^^,^ 31 Therefore, variability in rates of reported depressive symptoms exists, which could be explained by the use of different tools or various stages of PD.

Some findings differed to those of our study. The results reported by Serrano- Dueñas et al. found that the rates of depression in PD were higher in patients whose dominant side was primarily affected (p<0.001).^21^ Errea and Ara noted an association among females; 19 it is also reported that PD patients who are single exhibit a higher prevalence of depression^32^ as do those with the akinetic-rigid phenotype, which could be explained by the more severe neuronal depletion in the ventral tegmental area.^33^

On the other hand, factors that continued to be associated on the multivariate analysis were hyposmia, progression of disease, long-term use of L-dopa (greater than 4 years), and use of MAOIs, similar results to those found by Pinto L et al.^22^ It should be noted that in the present study, hyposmia was not evaluated objectively; however, many questionnaires reported the condition in their list of questions about non-motor symptoms experienced within the last month.^22^^,^ 25 ^,^ 27 ^,^ 28 It is well-known that olfactory loss can precede symptoms shared by both PD and Alzheimer’s disease by several years; it is argued that lack of awareness of hyposmia correlates with the presence of cognitive impairments of these degenerative disorders, a query that was omitted in this study because patients with cognitive impairment were excluded^34^^,^ 35 based on MMSE score.

Furthermore, the influence of MAOI use as a confounding factor could not be established; it has a clear relationship with symptoms of depression and cognitive performance^36^ and thus represented a major limitation of the study. In the ADAGIO study, treatment with rasagiline was reported to improve mood symptoms and the non-motor experiences of daily living.^37^^,^ 38 In addition, results from a small randomized, double-blind, placebo-controlled study have also suggested that rasagiline may exert beneficial effects on attention and executive abilities in non-demented PD patients with cognitive impairment.^39^ The ACCORDO study was a 12-week, double-blind, placebo-controlled trial to evaluate the effects of rasagiline 1 mg/day on depressive symptoms and cognition in non-demented PD patients with depressive symptoms. The primary efficacy variable was the change from baseline to week 12 in depressive symptoms measured by the Beck Depression Inventory (BDI-IA) total score. The treatment with rasagiline had no significant effects versus placebo on depressive symptoms or cognition in PD patients with moderate depressive symptoms. Although limited by lack of correction for multiple comparisons, post hoc analyses indicated some improvement in patient-rated cognitive and depression outcomes.^36^ In this study, the results failed to show an association with dopaminergic agonists (p=0.327). However, the only anti-parkinsonian therapy that has been shown in a randomized controlled trial to improve depressive symptoms in PD is pramipexole (patients in the pramipexole trial had lower baseline BDI scores of 18.7-19.5),^40^ which has been shown to bind with high affinity to dopamine D3 receptors in the prefrontal cortex, amygdala, and medial and lateral thalamus (all known to have some relationship with depression).^41^

Our results confirm the findings of Errea and Ara, indicating an association of the duration of the disease and lower cognitive performance with depression in PD patients.^19^ The finding also corroborates the results of the study of Wichowicz et al.^30^ in a Polish population, where patients with PD and depression were associated with a high UPDRS score, a high severity score on the HY scale, longer duration of PD, higher doses of equivalents of L-dopa, as well as older patients, more general impairment and greater clinical fluctuations. The present results are also consistent with those of Rodriguez-Violante et al.,^31^ who reported that the early onset of PD, greater severity of PD and a rapid progression of motor symptoms are risk factors for developing depression, as were the United Parkinson’s Disease Rating Scale (UPDRS) score and HY PD staging scale. Several studies found that depression in PD is associated with greater cognitive decline and increased motor symptoms.^3^ Chana-Caves et al., however, reported that depression in PD is associated with patients with non-motor symptoms who are non-responders to treatment with L-dopa,^29^ whereas the study by Bertucci et al. found that depression in home early-onset PD patients did not affect the disease significantly.^42^ All these findings show that the development of depression in PD is associated with a greater degree of severity and progression of the disease. It is possible that both diseases modify or condition one another as to their manifestation or progression. In order to avoid confusion in the interpretation of the results, we excluded patients with an MMSE <25, as depression could coexist with cognitive impairment in some patients. The pathophysiological basis for cognitive impairment and depression has been associated with a loss of serotonergic neurons in the raphe nuclei.^43^ Another study limitation is that we excluded patients with cognitive impairment based on the MMSE scale; a brief cognitive function test that quickly evaluates visuospatial functioning but is unable to evaluate executive function, while cognitive ability is evaluated by the MoCA;^44^ therefore, some study participants may have had undetected cognitive impairment; as the initial clinical factors of cognitive impairment in PD affect executive and visuospatial functions alike.^45^ Another limitation involves the lack of a scale to assess apathy in the cohort; where evidence shows that depression and apathy can coexist in a single patient or manifest independently, possibly attributable to loss of interest and/or motivation as a key factor in both. In fact, symptoms exist that are commonly shared by both depression and apathy, such as: psychomotor delays, anhedonia (inability to experience pleasure), anergia, decline in daily physical activity, and decreased enthusiasm for daily routines.^46^

Many of the studies report the frequency of depression when utilizing a test that incorporates depression into its questionnaire, such as the NMSQuest Scale, which can overestimate or underestimate the presence of depression in patients with PD. It is therefore recommended to use specific diagnostic tools to detect the presence of depressive symptoms in patients with PD. Our study assessed the presence of depression by using the BDI-II. This instrument is validated in the Spanish language, but not at the local level. It is thus important to validate its use in Peru for further investigations.

An important finding in our study was that only 20.6% of patients were receiving antidepressants, a figure much lower than those reported in other studies.^19^ Considering that depression is very common in patients with PD, it should be identified at an early stage in order to initiate timely pharmacologic and neuropsychological therapy, both proven to promote improvements in verbal memory and executive function.^47^

In conclusion, the factors associated with symptoms of depression in patients with PD were hyposmia, rapid progression of the disease, long-term use of L-dopa in years, and the use of MAOIs. The frequency of depressive symptoms in patients with PD was 60.9%.
